# Pharmacological and Psychosocial Strategies in the Management of Severe Behavioral Problems in Autism Spectrum Disorder: A Case Study

**DOI:** 10.1192/j.eurpsy.2026.11130

**Published:** 2026-07-31

**Authors:** I. M. Peso Navarro, C. Garcia Cerdan, M. Ligero Argudo, C. Munaiz Cossio, M. Covacho Gonzalez, N. M. Casado Espada

**Affiliations:** 1 Psiquiatria; 2 Complejo Asistencial Universitario de Salamanca, Salamanca, Spain

## Abstract

**Introduction:**

Autism Spectrum Disorder (ASD) is frequently associated with emotional dysregulation, behavioral disturbances, and sensory hypersensitivity, which may significantly impair family dynamics and academic adaptation. Pharmacological management is often challenging due to heterogeneous responses and paradoxical reactions to psychotropic medications.

**Objectives:**

To present a complex case of a child with ASD and severe behavioral dysregulation, highlighting the challenges of psychopharmacological treatment, the role of sensory processing difficulties, and the importance of a multimodal therapeutic approach.

**Methods:**

Case report based on longitudinal follow-up in child and adolescent psychiatry. Clinical information was collected through medical records, parental interviews, and collaboration with neuropediatrics, psychology, and educational services. Evolution under different psychopharmacological strategies (risperidone, aripiprazole, quetiapine, clonidine, guanfacine, haloperidol) and behavioral interventions was analyzed.

**Results:**

The patient exhibited severe agitation, physical aggression, and hypersensitivity to auditory stimuli, with episodes of emotional disconnection and rigidity. Multiple pharmacological trials showed limited efficacy or paradoxical reactions. Introduction of guanfacine, in combination with low-dose haloperidol, improved behavioral regulation and affective reciprocity. Psychotherapeutic work with the family emphasized psychoeducation, structured routines, and regulation strategies. Recent follow-up revealed improved affective expression, reduced aggression, and partial stabilization of sleep and feeding difficulties, although sensory triggers remain a challenge.

**Conclusions:**

This case illustrates the complexity of managing behavioral dysregulation in ASD, where comorbid sensory hypersensitivity and variable pharmacological responses require individualized and multimodal strategies. Combined pharmacotherapy, psychoeducation, and environmental adaptations can yield meaningful improvements, despite persistent vulnerabilities.

**Disclosure of Interest:**

None Declared

